# Dehydroascorbic Acids-modified Polymer Micelles Target Cancer Cells to Enhance Anti-tumor Efficacy of Paclitaxel

**DOI:** 10.1038/s41598-017-01168-7

**Published:** 2017-04-20

**Authors:** Xiaoyu Pei, Feifei Luo, Jun Zhang, Wulian Chen, Chen Jiang, Jie Liu

**Affiliations:** 1 Department of Digestive Diseases, Huashan Hospital, Fudan University, Shanghai, 200040 China; 2 Biotherapy Research Center, and Institute of Biomedical Sciences, Fudan University, Shanghai, 200032 China; 3 State Key Laboratory of Molecular Engineering of Polymers, Department of Macromolecular Science, Fudan University, Shanghai, 200433 China; 4 Key Laboratory of Smart Drug Delivery, Ministry of Education, Department of Pharmaceutics, School of Pharmacy, Fudan University, Shanghai, 201203 China

## Abstract

Paclitaxel (PTX), especially albumin-bound PTX in clinical, has displayed significant inhibition of tumor growth in patients. But the systemic distribution and poor water solubility of PTX often lead to severe side effects, consequently limiting the anti-tumor efficacy. In this study, we developed a novel PTX-loaded polymeric micelle drug delivery system. These self-assembled polymeric micelles from core to outside consisted of poly L-phenylalanine (pPhe), DTSSP linked poly L-lysine (pLys), poly ethylene glycol (PEG) and dehydroascorbic acids (DHA). pPhe formed the hydrophobic core to encapsulate PTX; DTSSPs on pLys covalently cross-linked and formed disulfide bond to stabilize PTX from loss in blood circulation; PEG improved solubility to lower toxicity of PTX for its high hydrophilicity; DHA targeted tumors by specifically recognizing GLUT1 mainly expressed on tumor cells. Thus, PTX would be precisely released into tumor cells with high dose of glutathione to break disulfide bond. Moreover, these PTX-loaded polymer micelles significantly suppressed tumor cell viability, proliferation, and migration *in vitro*, and also greatly inhibited tumor growth and prolonged survival in tumor-bearing mice without detectable side effects. Therefore, the new drug delivery system could reduce severe side effects and enhance anti-tumor efficacy of PTX via peripheral stabilization, low toxicity and tumor targeting.

## Introduction

Paclitaxel (PTX), especially albumin-bound PTX for cancers in clinical, has shown high effectiveness and broad-spectrum applicability^1–6^. The anti-cancer mechanisms of PTX mainly act on microtubules and combine with cellular tubulin to induce tubulin polymerization, prevent microtubule depolymerization and spindle formation, and make tumor cells arrest at M/G2 phases^7^. However, PTX causes systemic toxicity since it lacks tumor targeting. Additionally, because of the poor water solubility of PTX, polyoxyethylated castor oil (Cr emophor^®^EL) and ethanol have served as solvents to enhance its solubility. The solution also needs to be further diluted and filtrated in the solubilizer before used, while the solubilizer polyoxyethylene castor oil may cause the release of histamine, which leads to acute allergic reactions, even death^8^. Therefore, the development of a novel and low toxic formulation of paclitaxel has become a research hotspot in the field of medicine and pharmacology^9, 10^.

Polymeric micelles are burgeoning as a promising nanocarrier for anti-cancer therapy, as they improve solubility, safety, effectiveness, and bioavailability of hydrophobic anticancer drugs due to their well-defined core-shell architecture^11, 12^. In small sizes, polymeric micelles can accumulate passively in solid tumor sites by enhanced permeability and retention (EPR) effect, which is a unique anatomical pathophysiological nature of tumor blood vessels and lymphoid tissues^13^. In addition, through structural modification, they can stabilize the encapsulated drugs in blood circulation as well as ensure their accurate internalization and release in target tumor cells. Moreover, the hydrophilic shell of polymeric micelles can be grafted with various targeting ligands that recognize cancer cells to further enhance the active targeting and cellular internalization. Dehydroascorbic acid (DHA) is often employed to functionalize the polymeric micelles for active tumor targeting^14, 15^. It has a high affinity for glucose transport protein 1 (GLUT1)^16, 17^, which is selectively over-expressed on the surface of hepatic carcinoma cells (HepG2) and colon cancer cells (SW480)^16, 18–23^. As targeting ligands, DHAs are modified on the outside surface of the polymeric micelles, which leads to receptor-mediated targeted drug delivery^24^. In addition, the disulfide bond covalent cross-linking could enhance the stability of polymeric micelles in blood circulation and prevent drug unnecessary loss^25, 26^. The breakage of disulfide bonds and intracellular drug release can be triggered by the high level of glutathione (GSH) in the cytoplasm as compared with extracellular environment^27, 28^, which disassembles the polymeric micelles and subsequently releases PTX into cellular compartments.

**Figure 1:**
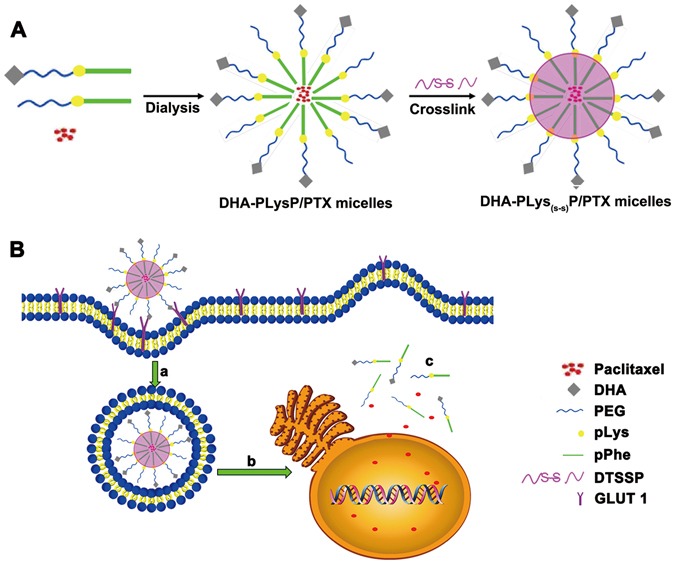
The development of PTX-loaded targeted cross-linked polymeric micelles.

In this study, we developed a novel PTX delivery system, which consisted of polymeric micelles modified by PEG to improve solubility, DHA to target tumors and disulfide bond covalent cross-link to stabilize PTX in blood circulation (Fig. 1). As a result, PTX could be delivered into tumor site and released only in tumor cells to display enhanced anti-tumor efficiency without severe side effects. Thus, our system might facilitate the clinical use of nano-carriers such as polymeric micelles in conquering cancers and other critical diseases.

## Results

### Synthesis of DHA-modified paclitaxel loaded polymer micelles

**Figure 2:**
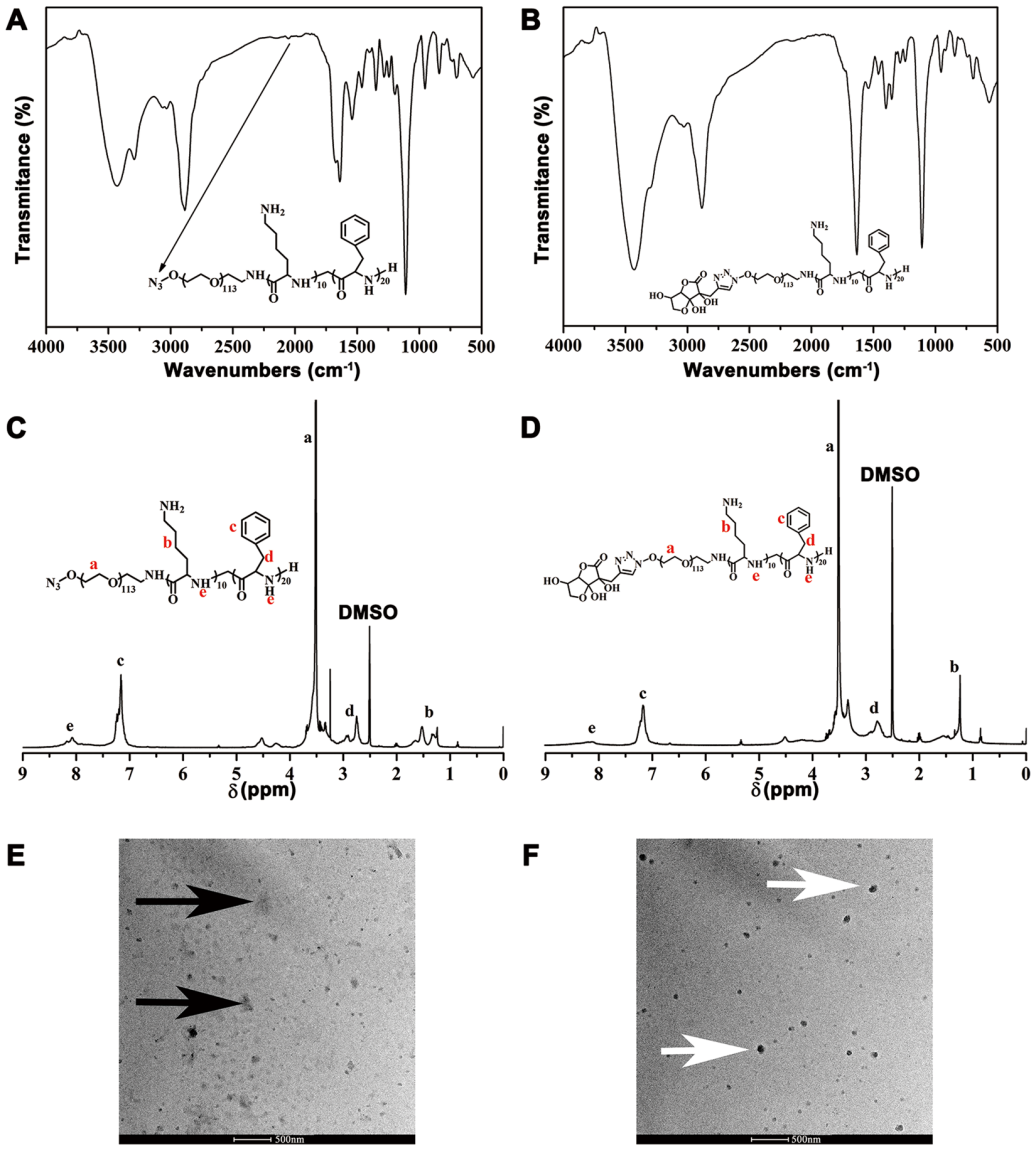
The characterization of the targeted and non-targeted copolymers. The FT-IR spectra of N^3^-PEG-pLys-pPhe (**A**) and DHA-PEG-pLys-pPhe (**B**). ^1^H NMR spectrum of N^3^-PEG-pLys-pPhe (**C**) and DHA-PEG-pLys-pPhe (**D**), in which the letters a–e correspond to the protons in the structural formula. TEM image of N^3^-PEG-pLys-pPhe (**E**, black arrows) and DHA-PEG-pLys-pPhe (**F**, white arrows).

The composition and chemical structure of the non-targeted and targeted amphiphilic triblock copolymers, N^3^-PEG-pLys-pPhe and DHA-PEG-pLys-pPhe, were confirmed by FT-IR and ^1^H NMR (Fig. 2). As shown in Fig. 2A, the peak of the group N^3^ appeared around 2030 cm^−1^ that was marked by the long arrow, whereas, this peak disappeared in Fig. 2B indicating that the targeted group DHA replaced the group of N^3^ in the structure of copolymer. The resonance peaks of protons were illustrated in the ^1^H NMR spectra of N^3^-PEG-pLys-pPhe (Fig. 2C) and DHA-PEG-pLys-pPhe (Fig. 2D). The morphologies of these two copolymers were observed by TEM (Fig. 2E and F). The targeted copolymers were more compact than the non-targeted ones visualized by TEM, as a result of the increased electrostatic repulsion after connecting the targeted molecules DHA. The Mn of N^3^-PEG-pLys-pPhe and DHA-PEG-pLys-pPhe calculated by Gel permeation chromatography (GPC) analyses were 11,300 g/mol and 12,100 g/mol, respectively. It is known that the Mn of polymeric compounds is a considerable value and the result of GPC detection showed the relative molecular weight distribution. Herein, either the group of N^3^ or DHA would not have great impact on the relative molecular weight distribution. These results demonstrated that the conversion and polymerization of amphiphilic triblock copolymers was successful.

### Characterization of DHA-modified paclitaxel loaded polymer micelles

As illustrated in Table 1, DHA-PLys_(s‑s)_P/PTX micelles exhibited a size of 106 nm and the zeta potentials of 29.77 mV after cross-linking, which were both significantly low compared to those of DHA-PLysP/PTX. This suggested that cross-linked modification reduced the intermolecular electrostatic repulsion and distance by decreasing primary amino groups. In addition, there was no major change in the PDI of these two structured polymeric micelles. This data indicated that after cross-linking, the targeted polymeric micelles became much more condensed and stable. PTX loading capacity was measured by HPLC. The drug loading content and drug encapsulation efficiency of DHA-PLys_(s‑s)_P/PTX was 90% and 4.71%, respectively, which was similar to that of DHA-PLysP/PTX.

**Table 1:** Particle size, polydispersity index and zeta potential of PTX-loaded polymeric micelles.

PTX-loaded polymeric micelles	Particle size (d, nm)	PDI	Zeta potential (mV)
PLysP/PTX	116.66 ± 0.19	0.18 ± 0.02	38.9 ± 0.80
PLys_(s-s)_P/PTX	98.27 ± 1.14	0.21 ± 0.06	30.57 ± 2.32
DHA-PLysP/PTX	125 ± 3.24	0.19 ± 0.01	40.8 ± 2.40
DHA-PLys_(s-s)_P/PTX	106.6 ± 3.02	0.20 ± 0.05	29.77 ± 1.32

### DHA-modified paclitaxel loaded polymer micelles inhibit proliferation and induce apoptosis in HepG2 and SW480 tumor cells

**Figure 3:**
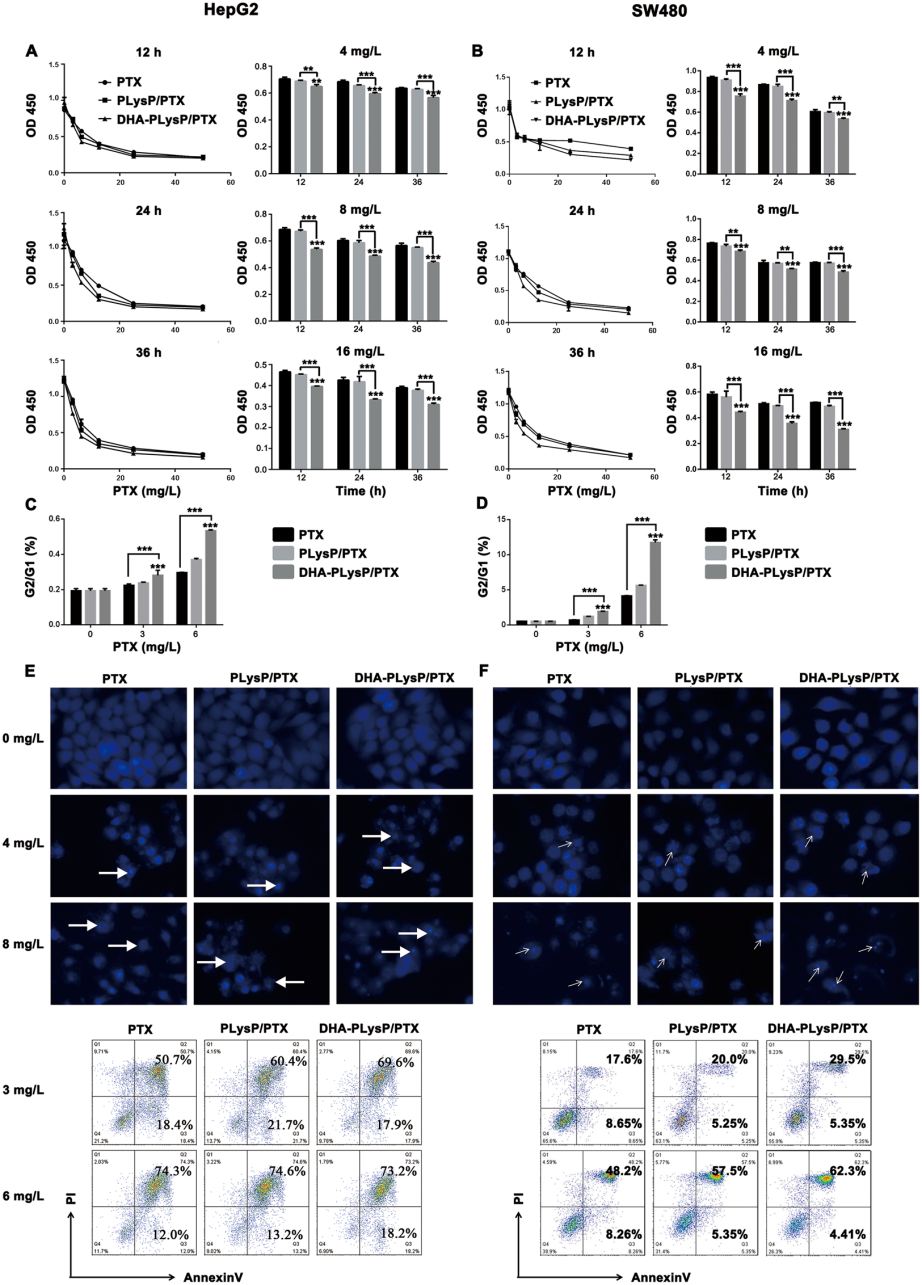
Proliferation inhibition and apoptosis induction of the HepG2 and SW480 cells under the treatment of free PTX, PLysP/PTX or DHA-PLysP/PTX. (**A,B**) The proliferation of HepG2 and SW480 cells under the treatment of PTX, PLysP/PTX and DHA-PLysP/PTX micelles with different dose of PTX for different time were detected at OD 450 nm. Six replicates each. (**C,D**) HepG2 and SW480 cells were treated with PTX, PLysP/PTX and DHA-PLysP/PTX micelles for 24 h, and then were stained by propidium iodide and analyzed by flow cytometry. The ratio of cells in G2 phase to those in G1 phase was shown. (**E,F** upper) HepG2 and SW480 cells were treated with PTX, PLysP/PTX and DHA-PLysP/PTX micelles at the final PTX concentration of 4 mg/L and 8 mg/L for 24 h and stained with DAPI to check the morphological changes of nucleus (magnification × 200). Nuclear fragmentation and chromatin condensation are indicated by white arrows. (**E,F** lower) HepG2 and SW480 cells were treated with PTX, PLysP/PTX and DHA-PLysP/PTX micelles at the final PTX concentration of 3 mg/L and 6 mg/L for 24 h and then were stained by Annexin V and PI before subjected to FACS for cell apoptosis analysis. **p < 0.01, ***p < 0.001.

In order to evaluate the effects of free PTX, PLysP/PTX and DHA-PLysP/PTX micelles on the proliferation of HepG2 and SW480 cell lines, the OD values were measured at 450 nm following 12, 24 and 36 h of treatment with different final concentrations of PTX. The dose-dependent and time-dependent growth inhibitory effects on the HepG2 and SW480 cells are shown in Fig. 3A and B. All of this data illustrated that proliferation inhibition ratios increased in a stepwise fashion following 12, 24 and 36 h of treatment and with 3.125, 6.25, 12.5, 25 and 50 mg/L final concentrations of PTX in all the three experimental groups. The difference in the OD values was statistically significant between both DHA-PLysP/PTX groups and control groups (P < 0.01), as well as between DHA-PLysP/PTX groups and PLysP/PTX groups (P < 0.01). And the IC 50 values of PTX, PLysP/PTX and DHA-PLysP/PTX were calculated and listed in Table 2. These results indicated that a higher final concentration of PTX was more potent than a lower one in terms of cytostatic effects, as well as that a longer duration of treatment was more potent than a shorter duration. Moreover, among the three PTX formulations, the DHA-PLysP/PTX micelles were the most efficient at anti-proliferation.

**Table 2:** IC50 values of different PTX formulations against HepG2 or SW480 tumor cells

Formulations	HepG2	SW480
PTX	10.466 mg/L	11.413 mg/L
PLysP/PTX	8.045 mg/L	8.978 mg/L
DHA-PLysP/PTX	5.041 mg/L	6.481 mg/L

Furthermore, by flow cytometric analysis, free PTX, PLysP/PTX and DHA-PLysP/PTX micelles were shown to induce cell cycle arrest at G2/M phase, and a significant dose-dependent increase in ratio of G1/G2 was observed as a result of the treatments in the HepG2 and SW480 cells (Fig. 3C,D). These indicated a blockage in the G2/M phase transition, which may lead to cell growth suppression or apoptosis.

DAPI staining assay (Fig. 3E,F upper panel) revealed that treatment with free PTX, PLysP/PTX and DHA-PLysP/PTX micelles resulted in marked nuclear fragmentation and condensation compared with the controls, suggesting more cell apoptosis. Moreover, the apoptotic body formation and nuclear fragmentation observed in the cells treated with DHA-PLysP/PTX micelles was significantly more than that in the PLysP/PTX and free PTX groups. Apoptosis-inducing effects of free PTX, PLysP/PTX and DHA-PLysP/PTX micelles were assessed by Annexin V and PI staining followed by flow cytometric analysis 24 h after treatment. As shown in Fig. 3E and F lower panel, the percentage of apoptotic cells in DHA-PLysP/PTX groups at the final PTX concentrations of 3 mg/L and 6 mg/L was higher than that in the free PTX and PLysP/PTX groups. Additionally, the PTX formulations effectively induced cell apoptosis in a dose-dependent manner.

### DHA-modified paclitaxel loaded polymer micelles suppress migration in HepG2 and SW480 tumor cells

**Figure 4:**
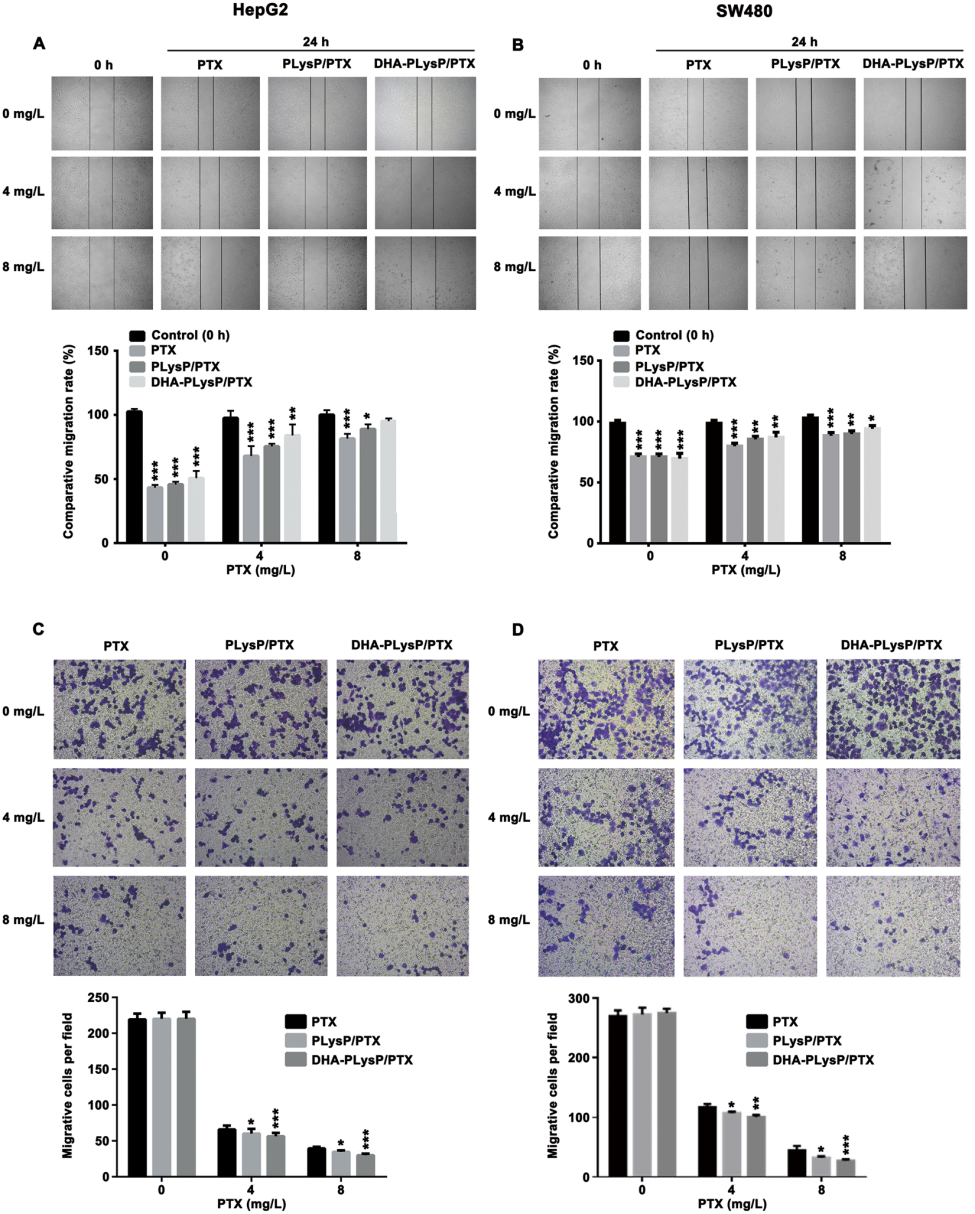
Migration inhibition of the HepG2 and SW480 cells under the treatment of free PTX, PLysP/PTX and DHA-PLysP/PTX micelles with different dose of PTX. (**A,B**) Differential cell migration ability was examined by the wound-closure assay (magnification × 100). Densitometric analysis on wound closure speed is expressed as % of control. (**C,D**) Representative statistical chart for the transwell assay showed a significant decrease in migration by DHA-PLysP/PTX micelles, relative to control. *p < 0.05, **p < 0.01, ***p < 0.001.

To detect the effect of free PTX, PLysP/PTX and DHA-PLysP/PTX micelles on the migration of HepG2 and SW480 cell lines, HepG2 and SW480 cells were exposed to the free PTX, PLysP/PTX and DHA-PLysP/PTX micelles, as well as subjected to wound healing assay and transwell migration assay. The results indicated that these three PTX formulations inhibited scratch wound closure (Fig. 4A,B). The wound gaps in the DHA-PLysP/PTX micelles groups were close to those in the control groups, which indicated that DHA-PLysP/PTX micelles significantly inhibited the migration of cancer cells, relative to the free PTX and PLysP/PTX micelles groups. Transwell migration assay similarly revealed that the migratory capacity of the HepG2 and SW480 cells was strongly inhibited by DHA-PLysP/PTX micelles compared with free PTX and PLysP/PTX micelles (Fig. 4C,D). Simultaneously, the effect of the PTX formulations on cell migration obeyed a dose-dependent manner.

### Anticancer effect of DHA-modified paclitaxel loaded polymer micelles in HepG2- and SW480-bearing mice

**Figure 5:**
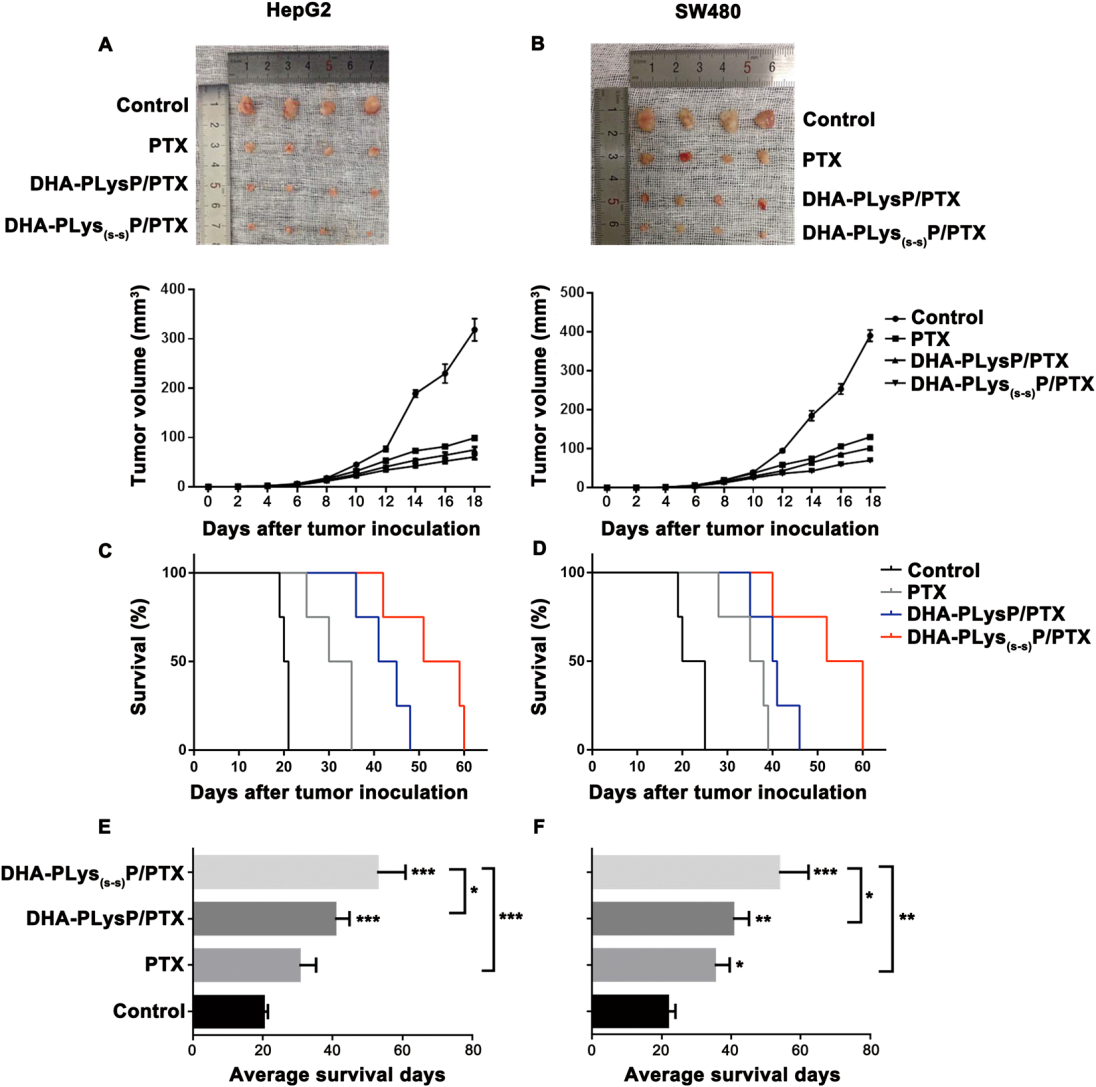
Effects of free PTX, DHA-PLysP/PTX and DHA-PLys_(s-s)_P/PTX micelles on HepG2- and SW480-bearing nude mice. Thirty-two female nude mice were implanted *s.c*. with 1 × 10^6^ HepG2 or SW480 cells. Approximately 5 days after the implantation, these tumor-bearing nude mice were *i.v*. treated with different PTX formulations every three days for total five times. (**A,B**) On day 18, 16 tumors from different treatment groups were excised (n = 4). The tumor sizes were measured with a digital caliper every two days. (**C,D**) Mice survival was monitored regularly. Log-rank test (n = 4). (**E,F**) The average tumor volumes were analyzed (n = 4). *p < 0.05, **p < 0.01, ***p < 0.001.

To further verify the above findings, anti-tumor efficacy of free PTX, PLysP/PTX and DHA-PLysP/PTX micelles were tested in HepG2- and SW480-bearing nude mice. During the treatment period, no mice died in any of the groups. The tumor volumes were monitored and recorded for 18 days. As shown in Fig. 5A and B, both PTX-loaded targeted micelles showed obvious anticancer efficacy; the crosslinked group showed better tumor inhibition effects than non-crosslinked group. Although the free PTX was efficacious, the weight loss of cancer-bearing mice to some extent indicated the systemic toxicity of PTX^29^. Compared with free PTX and the non-crosslinked groups, the mental status and activities of mice in the crosslinked group did not signify any abnormal behavior. As shown in Fig. 5C–F, the survival time was prolonged after treatment with the DHA-PLysP/PTX and DHA-PLys_(s-s)_P/PTX micelles, and the best results were also obtained from the group with crosslinked micelles, which was consistent with the previous tumor inhibition results. According to these comparative experiments, the survival days were markedly prolonged in the DHA-PLys_(s‑s)_P/PTX treated group and this group achieved the greatest antitumor efficacy. Therefore, DHA-PLys_(s‑s)_P/PTX micelles may have great potential in cancer chemotherapy, owing to their ability to accumulate PTX in targeted site through increasing the stability of drug-loaded micelles and decreasing drug leakage.

## Discussion

Polymeric micelle-based drug delivery systems present a promising strategy for an efficient cancer-targeting therapy^30, 31^. In this study, we evolved novel PTX-loaded polymeric micelles modified by PEG to improve solubility, DHA to target tumor and disulfide bond to stabilize PTX but release it in tumor cells^32^, consequently adverse reaction is reduced and chemotherapy efficacy is improved. The core-shell shaped polymeric micelles were obtained by entrapping PTX in the PEG-pLys-pPhe copolymer, which was biodegradable and biocompatible. PEG is considered safe in the body for its high hydrophilicity, high biocompatibility, low toxicity, and low immunogenicity. Moreover, pLys and pPhe are biodegradable polypeptides widely used in biomedical fields. These polymeric compounds can be applied for graft-modification, and further used in wide range of applications^33, 34^. The modification of DHA ligands and covalent cross-linked disulfide bonds can not only endow the active targeting for cancer cells and tissues, but also strengthen stability and avoid drugs leaking in the blood circulation, which reduces the toxicity to non-target tissues and increases safety of PTX-loaded polymeric micelles *in vivo* use simultaneously^27, 35, 36^.

In order to further investigate and validate the cancer targeting, safety and efficacy of PTX-loaded targeted polymeric micelles, we examined and compared the anticancer effect through *in vivo* and *in vitro* experiments. Our data demonstrated that the DHA-PLysP/PTX micelles had stronger ability to inhibit cancer cell growth and migration, as well as promote cancer cells apoptosis than free PTX and PLysP/PTX micelles, which illustrated that the targeting molecule DHA was able to recognize tumor cells specifically. This is consistent with the report that DHA was considered as a potential small molecule for tumor-specific recognition and transportation^37, 38^, and PTX-loaded polymeric micelles achieved positive-targeting transportation under the mediation of DHA. We also found using *in vivo* experiments that DHA-PLys_(s-s)_P/PTX micelles, as opposed to free PTX and DHA-PLysP/PTX micelles, could effectively inhibit tumor volumes and weights of tumor-bearing nude mice, and prolong survival times without serious side effects. These results support the idea that disulfide bonds, as an anti-leakage barrier^28^, improved the stability of PTX-loaded targeted polymeric micelles in the blood circulation to reduce drug leakage in peripheral blood system and increase drug accumulation in the tumor sites.

To develop and advance the clinical application of these PTX-loaded targeted polymeric micelles, further studies should employ human primary tumor models to study the anticancer effect. Since they maintain the global gene-expression patterns, histologic architecture, molecular signatures, and drug responsiveness of the original patient tumors, human primary tumor models may provide a more reliable response of human tumor biological characteristics to the PTX-loaded targeted polymeric micelles than cell-line xenograft models^39, 40^. On the other hand, these studies could focus on simplifying the preparation of drug-loaded polymeric micelles and optimizing structural modification. The appropriate technology improvements could be propitious to scaled production of PTX-loaded targeted polymeric micelles for both next step experimental study and future medicine production.

In summary, through *in vivo* and *in vitro* studies, it was demonstrated that these novel PTX-loaded polymeric micelle formulations have the advantages of low toxicity, target specificity and high efficiency for cancer therapy. Therefore, they could be expected to become safe and effective tumor-targeted chemotherapy agents and be used in clinical.

## Methods

### Cell lines

The human hepatic carcinoma and colon carcinoma cell lines, HepG2 and SW480, were purchased from the cell bank of Chinese Academy of Sciences. These two kinds of tumor cells were grown as adherent cultures in DMEM (Gibco, USA), supplemented with 10% fetal bovine serum (Gibco, USA) and 1% penicillin-streptomycin solution (Gibco, USA) under the atmosphere of 5% CO_2_ humidified conditions at 37 °C. These cells were fed until confluence and digested by 0.25% trypsin (Gibco, USA). All cellular experiments were performed under the exponential growth phase of the cells.

### Animals

Female BALB/c nude mice, weighing 18–20 g and aged 5–6 weeks, were purchased from Slac Experimental Animals Co., Ltd (Shanghai, China). All animals were raised in compliance with guidelines under specified pathogen-free (SPF) conditions. All animal experiments were performed in accordance with the Guidelines for the Care and Use of Laboratory Animals (No. 55 issued by Ministry of Health, China on January 25th, 1998), and all experimental protocols were approved by the Institutional Animal Care and Use Committee of Fudan University (20150493A177).

### Synthesis of DHA modified polymeric copolymers

**Figure 6:**
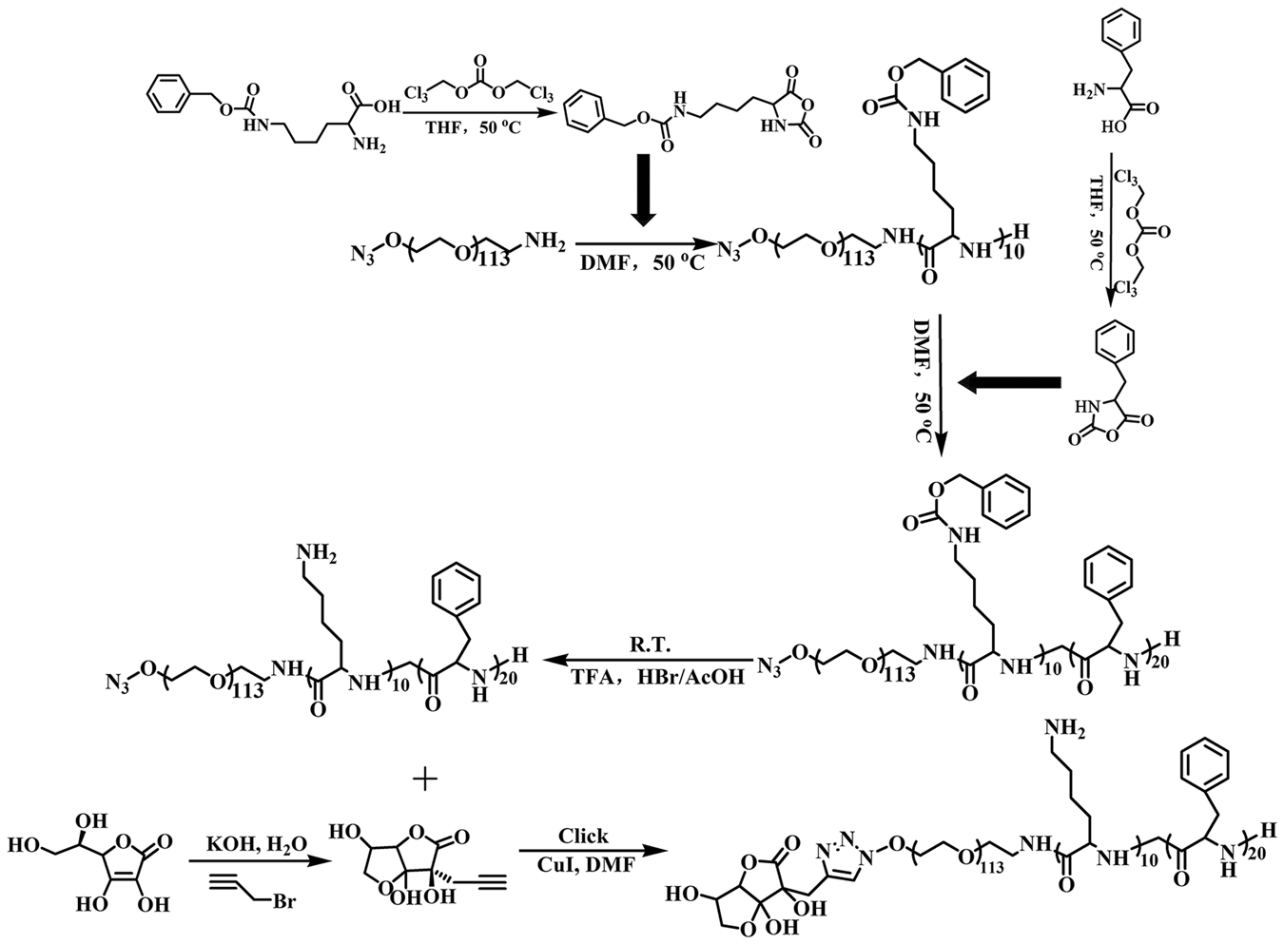
Synthetic route of DHA modified polymeric copolymers.

The synthetic route is shown in Fig. 6. Briefly, N^6^-Carbobenzyloxy-L-lysine N-carboxyanhydride (Lys (Z)-NCA) (J&K Scientific, China) and L-phenylalanine N-carboxyanhydride (Phe-NCA) (J&K Scientific, China) were synthesized according to the Fuchs-Farthing method using diphosgene^41^. The reaction equations are shown at the top left and right of Fig. 6. Subsequently, a stirred solution of N_3_-PEG-NH_2_ (1 g, 0.2 mmol) (JenKem, China) in anhydrous N,N-Dimethylformamide (DMF, 15 mL) (Sinopharm, China) was added to Lys(Z)-NCA (736 mg, 2.4 mmol) at 35 °C under nitrogen gas. After 24 h, Phe-NCA (917 mg, 4.8 mmol) and DMF (15 mL) were added to the mixture, and the reaction was maintained at 35 °C for a further 48 h. The copolymer mixture was isolated by repeated precipitation from DMF into diethyl ether and dried at room temperature (Yield: 90%). The hydrolysis of N^3^-PEG-pLys(Z)-pPhe was then performed by treating the block copolymer with trifluoroacetic acid (10 mL) and HBr/HOAc (0.5 mL) to remove benzyl groups. Finally, the aqueous solution of N^3^-PEG-pLys-pPhe was dialyzed using a membrane bag (Molecular weight cut-off (MWCO): 1000) (Union Carbide, USA) at room temperature for 24 h, followed by freeze-drying to yield an off-white solid. The DHA modification to the terminus N^3^-PEG-pLys-pPhe was prepared by copper-catalyzed azide–alkyne cycloaddition (click reaction)^42^. First, we connected a propargyl group to the DHA. Under nitrogen gas, freshly prepared solution of CuI (0.5 mmol) and N, N-Diisopropylethylamine (1 mmol) were added into the mixed solution of N^3^-PEG-pLys-pPhe (500 mg, 0.1 mmol) and azidated DHA (43 mg, 0.2 mmol) (Sigma, USA) in DMF. The reaction was performed at 30 °C for 12 h. The product was transferred to a membrane bag (MWCO: 1000) to dialyze against edetic acid disodium salt (pH 7.0) for 24 h and deionized water for another 24 h, followed by freeze-drying.

### The loading of PTX and the cross-linking of the polymeric micelles

PTX-loaded polymeric micelles were prepared by the dialysis method and a subsequent cross-linking reaction^43^. N_3_-PEG-pLys-pPhe (10 mg) and PTX (purity > 99.9%, 0.5 mg) (Melonepharma, China) were dissolved in 0.5 mL of DMF. The solution was dialyzed using a membrane bag (MWCO: 1000) against deionized water. After 24 h, the non-targeted and non-cross-linked PTX-loaded micelles, PLysP/PTX, were collected. To prepare cross-linked micelles, the disulfide-containing cross-linking agent, DTSSP (Thermo, USA), was employed to drop into the aqueous solution of PTX-loaded micelles. The dialysis reaction was carried out for 24 h against phosphate buffer saline (PBS, pH 8.0) and deionized water for another 24 h to obtain the cross-linked polymeric micelles, PLys_(s‑s)_P/PTX. In order to fabricate the DHA-modified micelles, DHA-PLys_(s‑s)_P/PTX, 20 mole percentage (of total copolymers) of DHA-PEG-pLys-pPhe was added into the DMF solution of N_3_-PEG-pLys-pPhe and PTX. The rest process was the same as mentioned above.

### Characterizations of copolymers and micelles

Fourier transform infrared spectra (FT-IR) (Thermo, USA) were detected on a Nicolet 6700 spectrometer. Samples were grinded to powder with KBr and compressed into a transparent tablet. ^1^H Nuclear Magnetic Resonance (^1^H NMR) spectra were measured by 400 MHz FT-NMR Spectrometer (Bruker, Germany). Gel permeation chromatography (GPC) was used to analyze the molecular average weights of copolymers recorded by the gel permeation chromatographer (Agilent, USA). By using the principle of dynamic light scattering (DLS), the average sizes, PDI, and zeta potentials of micelles were determined via Nano-particle size and Zeta potential instrument (Malvern, UK). The morphological examination of copolymers was carried out by transmission electron microscopy (TEM) (Philips, USA). A UV spectrophotometer (Shimadzu, Japan) was used to determine the drug loading content and the drug encapsulation efficiency.

### CCK-8 proliferation assay

The activity of cell proliferation was assessed by Cell Counting Kit-8 (CCK-8) (Beyotime, China). Briefly, the cells in suspension were inoculated to a 96-well culture plate at a density of 10^4^ cells per well, and incubated for 24 h at 37 °C. Free PTX, PLysP/PTX or DHA-PLysP/PTX micelles were added into the wells. Six final concentrations of PTX (0 mg/L, 3.125 mg/L, 6.25 mg/L, 12.5 mg/L, 25 mg/L and 50 mg/L) comprised the concentration gradient to study the dose-dependent effect on cell viability. As for time-dependent cell proliferation assay, we chose three final concentrations of PTX (4 mg/L, 8 mg/L and 16 mg/L) and detected after incubation for 12 h, 24 h or 36 h, respectively. Each treatment had six parallel wells. After the incubation for the respective time, 10 μL CCK-8 reagent was added to each well, and together they were cultured in a 37 °C, 5% CO_2_ incubator for 2 h. Subsequently, the optical density values (OD values) were measured using a high-throughput enzyme-linked immunosorbent assay reader (Tecan, Switzerland) under 450 nm excitation wave lengths.

### Wound healing assay

Cells were cultured in 12-well plates until the confluence reached 80%. Similar sized wounds were made in monolayer cells with 200 μL sterile pipette ends, artificially. Free PTX, PLysP/PTX or DHA-PLysP /PTX micelles were added into 2.5 mM D-glucose cell-culture mediums at three concentrations (0, 4 or 8 mg/L). Each concentration set three parallel wells. The speed of wound closure was monitored and photographed after 24 h of incubation. Wound repair was assessed by measuring the remaining wound width. The width of fresh original wounds was set to ‘1’ and the width of the wound treatment groups were contrasted to it in order to calculate the relatedly migration rates. The comparative speed of wound closure of treated cells against the negative control was calculated using the following formula: Migration inhibitory rate (%) = 1 - (the wound width of treated cells at 0 hour - the wound width of treated cells at the 24th hour)/(the wound width of negative control cells at 0 hour - the wound width of negative control cells at the 24th hour) × 100%.

### Transwell cell migration assays

The migration assays were performed with 24-well Transwell chambers (Costar, USA). After starvation treatment, 1 × 10^5^ cells were added into the upper chamber of a transwell with cell-culture mediums (1% FBS, 2.5 mM D-glucose). Meanwhile, the two final concentrations of PTX (4 or 8 mg/L) of free PTX, PLysP/PTX or DHA-PLysP /PTX micelles were added into the upper chamber. The 2.5 mM D-glucose cell-culture mediums with 10% FBS were added to the lower chamber. After incubation for 12 h, cells that had migrated to the bottom of the filter were fixed by 4% Paraformaldehyde (PFA) and stained with 1% crystal violet. Each experiment was repeated three times. Cell counting statistics were carried out from eight randomly selected horizons per well under an inverted microscope (Zeiss, Germany) with 100 × magnification.

### DAPI nuclear staining

For DAPI nuclear staining assay, the tumor cells were prepared in 12-well cell culture plate at a density of 1 × 10^4^ cells/well and cultured until the confluence reached 80%. Free PTX, PLysP/PTX or DHA-PLysP/PTX micelles were added into 2.5 mM glucose culture medium with the final concentration of paclitaxel of 0, 4 or 8 mg/L (three parallel wells each). After 24 h of incubation, the cells were washed by PBS and fixed by 4% PFA for 30 min at room temperature. The fixed cells were stained by DAPI working solution in dark for 10 min. The nuclear morphology was observed and photographed using an inverted fluorescence microscope (Zeiss, Germany) with ultraviolet wavelength excitation. The magnification was 200×.

### Flow cytometric analysis of cell cycle and apoptosis

All cells were seeded at a density of 5 × 10^5^ cells/well in 6-well plates. After pre-balancing with glucose-free medium, the cells were incubated with free PTX, PLysP/PTX or DHA-PLysP /PTX micelles for 24 h with the final concentration of paclitaxel at 0, 3 or 6 mg/L. For cell cycle analysis, the cells were harvested and washed with cold PBS, and were then collected and stored at 4 °C overnight. The fixed cells were re-suspended in 0.5 mL propidium iodide (PI) staining solution and incubated in the dark for 30 min. The cell cycle distribution was detected by FACS calibar Flow cytometer (Millipore, USA) at an excitation wavelength of 488 nm. The percentage of cell cycle phases was calculated by FlowJo Software. Cell apoptosis was evaluated using an Annexin V-FITC cell apoptosis detection kit (Beyotime, China). According to the instructions, the cells were rinsed with cold PBS following incubation with free PTX, PLysP/PTX or DHAPLysP/PTX micelles for 24 h and then centrifuged at 1000 rpm for 5 min. The cell pellets were gently re-suspended in PBS and counted. Cell suspensions were prepared by 1× binding buffer. Each sample was then stained by Annexin V-FITC and PI in the dark. After 20 min of staining, the cells were immediately analyzed using FACS Calibur flow cytometer (Millipore, USA) and FlowJo Software to calculate the percentage of cell apoptosis.

### Establishment of cancer models in nude mice and treatment

The tumor cells were diluted in 0.9% physiological saline. The right flank of the nude mice was subcutaneously injected with 200 μL tumor cells (1 × 10^6^). After surgery, the nude mice were observed every two days. All of the mice recovered from the surgery, and the tumor formation rate was almost 100%. Approximately 5 days after the implantation, when the diameter of the tumor had reached about 1–3 mm, the tumor-bearing nude mice were randomly divided into 4 groups: control group (0.9% physiological saline, n = 8), free PTX-treated group (10 mg PTX/kg, n = 8), DHA-PLysP/PTX-treated group (10 mg PTX/kg, n = 8) and DHA-PLys_(s-s)_P/PTX-treated group (10 mg PTX/kg, n = 8). The drugs were administrated via fundus intravenous every three days for a total of five times. The general condition of the mice was monitored every other day. When the treatment was completed, four mice in each group were randomly separated and sacrificed by cervical decapitation. The subcutaneous tumors were dissected carefully and measured. The tumor size was measured with calipers for length (L, mm) and width (W, mm) every other day, and the tumor volume (V, mm^3^) was calculated using the formula: V (mm^3^) = 0.5 × L × W^2^, where L is the longest diameter and W the shortest width. The tumor weight inhibition rate (IR) was calculated according to the formula: IR (%) = (1-weight of treated group/weight of control group) × 100%.

The rest mice were raised for survival study. All mice were observed for a maximum post-implantation period of 60 days. In the event of the following two cases, the mice were considered dead: (i) the tumor size reached 10 mm × 10 mm; (ii) serious tumor complications occurred, e.g. ascites, tumor surface ulceration, severe weight loss.

### Statistical analysis

Analysis was performed by GraphPad Prism 6.0 software. The statistical significance of differences between experimental and control groups was determined using Student’s T-test. The comparative survival rates were analyzed by a log rank test. P-values less than 0.05 were considered significant, as demonstrated by asterisks in the Figures.
